# Joint Communication and Sensing: A Proof of Concept and Datasets for Greenhouse Monitoring Using LoRaWAN

**DOI:** 10.3390/s22041326

**Published:** 2022-02-09

**Authors:** Ritesh Kumar Singh, Mohammad Hasan Rahmani, Maarten Weyn, Rafael Berkvens

**Affiliations:** IDLab—Faculty of Applied Engineering, University of Antwerp—imec, Sint-Pietersvliet 7, 2000 Antwerp, Belgium; mohammad.rahmani@uantwerpen.be (M.H.R.); maarten.weyn@uantwerpen.be (M.W.); rafael.berkvens@uantwerpen.be (R.B.)

**Keywords:** artificial intelligence, ANN, greenhouse, LoRaWAN datasets, MLP, precision agriculture, smart farming, sensor network

## Abstract

In recent years, greenhouse-based precision agriculture (PA) has been strengthened by utilization of Internet of Things applications and low-power wide area network communication. The advancements in multidisciplinary technologies such as artificial intelligence (AI) have created opportunities to assist farmers further in detecting disease and poor nutrition of plants. Neural networks and other AI techniques need an initial set of measurement campaigns along with extensive datasets as a training set to baseline and evolve different applications. This paper presents LoRaWAN-based greenhouse monitoring datasets over a period of nine months. The dataset has both the network and sensing information from multiple sensor nodes for tomato crops in two different greenhouse environments. The goal is to provide the research community with a dataset to evaluate performance of LoRaWAN inside a greenhouse and develop more efficient PA monitoring techniques. In this paper, we carried out an exploratory data analysis to infer crop growth by analyzing just the LoRaWAN signals and without inclusion of any extra hardware. This work uses a multilayer perceptron artificial neural network to predict the weekly plant growth, trained using RSSI value from sensor data and manual measurement of plant height from the greenhouse. We developed this proof of concept of joint communication and sensing by using generated dataset from the “Proefcentrum Hoogstraten” greenhouse in Belgium. Results for the proposed method yield a root mean square error of 10% in detecting the average plant height inside a greenhouse. In future, we can use this concept of landscape sensing for different supplementary use-cases and to develop optimized methods.

## 1. Introduction

In the wake of recent global warming, greenhouse farming has helped farmers to reduce investment risks [1], led by precision agriculture (PA) technologies. It saves crops from extreme hail, heat, and wind along with providing a variety of solutions with optimal conditions to maximize quality yields and profitability. Greenhouse technology creates an ideal climate-controlled environment for plants or crops to ensure higher productivity with minimum labor costs. Greenhouse farming increases stability and security by enabling year-round farming with minimized production risk and more control against diseases.

In the greenhouse, environmental factors such as ventilation, light, humidity, and temperature can be controlled, allowing the farmer to create suitable micro-ecosystems for plants. Internet of Things (IOT)-enabled greenhouses achieve this by collecting data from various points inside the greenhouse at unprecedented granularity. An effective IOT solution for greenhouse incorporates a wireless sensor network (WSN) for processing and analyzing the data using cloud services. This provides new insights to the grower along with aligned recommendations for better decision-making. IOT-based evaluation of greenhouse before and during the crop growth assists growers to evaluate and adjust microclimate parameters. The main elements of a IOT-based data acquisition solution should be low power, exhibit accuracy, and support real-time processing. Internal and external conditions of the greenhouse impacts sensor, functional properties, and communication links as well [2]. A low-power wide area network (LPWAN) is the most optimal technology for agricultural operations inside a greenhouse [3]. A variety of LPWAN technologies exist, such as NB-IOT, Sigfox, and LoRaWAN. Among these, LoRaWAN is adapted on a higher scale due to its low cost, long-range communication, and low-power capabilities [4]. These technologies give flexibility to cover a vast distance with lower costs, but limit the frequency and amount of data transmission.

Typically, not all crops are suited for greenhouses, but those requiring a narrow range of environmental variables are well suited. For example, tomatoes are well suited to be grown inside greenhouses, as they need a great deal of attention. Tomatoes are highly susceptible to diseases such as powdery mildew, a fungal disease that can hamper the cultivated crops [5]. Another important fungal disease that impacts tomato crop, typically in Europe, is Oidium neolycopersici [5]. Infections are seen as powdery white lesions on the tomato leaves and spread by the dispersion of its spores. This impacts growth of a plant, fruit quality, and premature senescence. These diseases depend upon environmental conditions such as temperature, humidity, and light. Once it starts spreading, it is vital to diagnose as early as possible and apply chemical control using fungicides. Moving or isolating infected or diseased plants from the rest of the crop can help in prevention of crop disease throughout the greenhouse in limited time and space. Therefore, climate control methods are a fundamental segment of pest and disease management inside the greenhouse.

To support stringent monitoring of the greenhouse, not only does deployment of sensor network need to be exploited, but it is also imperative to find newer strategies to examine plant health in the greenhouse. One such strategy is to employ existing wireless sensing systems inside the greenhouse and tailor application actions accordingly. An example could be to use wireless signals of the existing communication infrastructure to detect several aspects of crop growth. Installation of radar for such a use case can provide a rich representation of environment. However, a typical IOT-based greenhouse solution does not have radar, and adding it would add heavy cost and complexity to the solution. Therefore, in this paper, we propose a proof of concept (POC) to predict weekly plant growth that can be used to derive other macroenvironmental features, such as crop health, using wireless communication signals only. Specifically, for this concept, we use received signal strength indicator (RSSI) of an LoRaWAN-based sensor network in the greenhouse to detect expected plant growth and answer the hypothesis questions such as prediction of weekly plant growth. In a greenhouse, it is very crucial to continuously analyze plant growth and accordingly manage the nutrients supply. Detecting plant growth using wireless signals, i.e., without adding any extra infrastructure, can have significant benefits such as aid in plant growth management. It can also enhance spatial representation of plants in the greenhouse to support KPI requirements.

### 1.1. Related Work

There exist some research contributions that used sensors and radars for environmental sensing. For example, to sense parking lots, Wu and Zwick [6] used sensing infrastructure to build a car park detection system. In these work scenarios, the application usually uses RSSI fluctuations in real time with path loss models to evaluate positioning. For example, Li et al. [7] proposed a method for indoor positioning based on RSSI distance model. Many positioning solutions such as GPS (Global Positioning System) have been used for outdoor applications. However, methods such as angle of arrival (AOA) and time of arrival (TOA) have been consequently used for effective indoor positioning. Paramount consideration is that the radio signals face interference and fluctuations caused by indoor obstacles. To handle this issue of solving complex RSSI estimation, many machine learning (ML) and artificial intelligence (AI) learning methods have been employed, such as neural network (NN) and fuzzy logic. Ahmadi and Bouallegue [8] carried out a comprehensive survey on ML techniques and RSSI for localization in WSN. It emphasizes RSSI-based sensing, since there is no need for additional hardware to measure this parameter. Another approach uses high-resolution maps along with Geographic Information System (GIS) to draw inference on landscape design [9].

With the increase in development of AI, the greenhouse sector has been able to change the dynamics of efficiency and precision. AI acts as a bridge between plants, growers, and an IOT or climatic control system inside a greenhouse. It helps the grower to identify a problem, forecast long-term issues, and harvest accordingly. Both researchers and industries are very active in optimizing and developing AI-based solutions for greenhouses. From an academic perspective, Wageningen University and Research (Netherlands) [10] is one of the leading academic institutes for AI-controlled cultivation. Hosts of companies such as Microsoft [11] and LetGrow [12] have been early adopters of AI for their commercially available solutions. Efficiency and cost are two primary features driving solutions to incorporate AI. Applications for greenhouses such as Luna by iUNU [13] use computer vision through AI to scan throughout the production area. It uploads images to analyze using different AI algorithms. Another solution, Bold Robotic Solution [14], works on AI systems using sensors to monitor and identify efficiency and production issues. It observes patterns to make corrections in controlling the equipment.

In our recent survey [15], we highlighted the significance of integrating multidisciplinary approaches towards the future of agriculture by proposing an “AgriFusion” architecture. It emphasizes that the optimal way to achieve agricultural demand is by integration of technologies such as AI and WSN in a unique system architecture. A comprehensive review on different sensor data analytics and ML algorithms is performed in [16], specifically on WSN using ML for forecasting and data mining. artificial neural network (ANN) does not rely on previous characteristics and assumptions to outline inputs and outputs. In a paper by Ghosal et al. [17], efficiency improvement of semiconductor gas sensors is achieved using an ANN model. Application of ANN is extending to greenhouse application. An article by Escamilla-García et al. [18] performs an extensive survey on different ANNss in greenhouses. Most of the study was focused on prediction of microclimate and highlights need for the hybrid model using ANN and physical models for a better solution. The work by P. Anjaiah [19] carried out an analysis of feedforward architecture for various greenhouse tasks. It presents many ANN applications in the context of greenhouse, majorly for prediction and variation of microclimate. This paper also emphasizes a major guideline for future work, by developing models to predict and manage the generated information. Another recent comprehensive review, by Sharma et al. [20], concludes that future scope in the agricultural sector is through an ML approach for sustainable use of available resources. It lists different multilayer perceptron (MLP) use-cases, for example, agricultural dataset tested with MLP algorithm, identification of cucumber virus through MLP neural network classifier, and harvesting application through object detection feature trained on an MLP. Another work by Castañeda-Miranda and Castaño [21] carried out frost control in the greenhouse using MLP in the central region of Mexico. Ref. Mavridou et al. [22] brings orientation of semistructured environments which are normally known to the grower, so that this information could be used by an ML algorithm for different use-cases, such as deterministic automation tasks and others related to them. Ref. Moon et al. [23] used MLP for interpolation of the greenhouse environment and demonstrated that MLP has the best performance among interpolation methods applied over greenhouse data. By an in-depth review of the state of the art, we observed that MLP was majorly used in a hybrid system such as using image and model-based features [24], thereby we took this as a motivation to use MLP for our work as discussed in Section 3.

### 1.2. Contributions

This paper presents LoRaWAN-based greenhouse monitoring datasets which can be used to develop different methods for PA. All datasets were collected in real greenhouse environments; no simulation models were used. Firstly, we created a dataset by deploying 27 sensors for a tomato greenhouse based in the research center Hoogstraten (PCH), Belgium. Thereafter, we deployed 19 sensors in another greenhouse in the Netherlands, again for the tomato crop. Secondly, we carried out the outliers identification and data analysis over collected datasets for building our POC. The datasets are published in Zenodo with the DOI 10.5281/zenodo.5793685.

**Figure 1:**
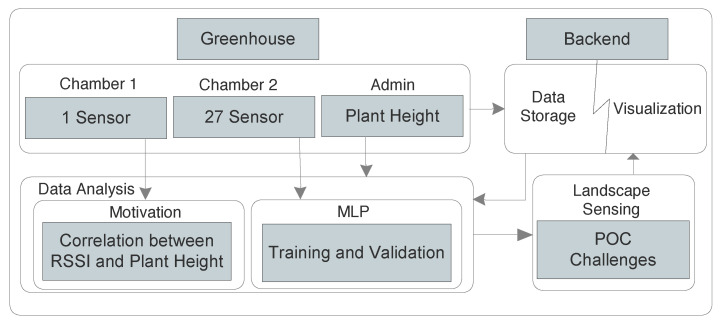
Figure illustrating the flow of experiments and data interaction to derive landscape sensing in the greenhouse. Data from chamber 1 is taken to set up the hypothesis, and chamber 2 data along with manual plant height data recorded from the admin in greenhouse is used for MLP.

Considering the above-discussed state-of-the-art, there is involvement of external support from entities, such as radars, sensors, etc., for sensing approach. This limits application specifically in the agricultural context precisely because of cost. To the best of our knowledge, this is the first POC which proposes a method where a grower can infer plant growth inside a greenhouse using only LoRaWAN signals, without really integrating any new sensor or infrastructure. We propose an AI-based MLP method to derive inference on plant growth by predicting the weekly plant height. To this end, we employed RSSIs as solely input features and weakly measurements of plant heights as labels for the supervised learning, as described in Figure 1. In addition, we further used the dataset to analyze LoRaWAN channel utilization, relation between temperature and humidity readings in the greenhouse, and impact of optimal number of sensors for sensing and their respective location for landscape sensing.

**Figure 2:**
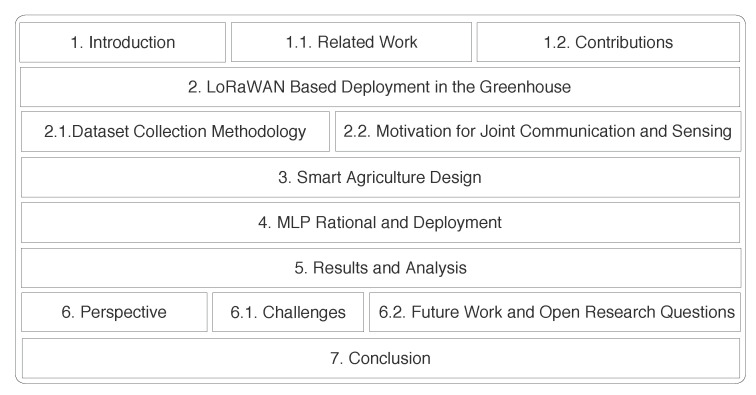
Structure of the paper.

The remainder of this paper is structured as shown in Figure 2. Section 2 describes deployment of the LoRaWAN-based sensors in the greenhouse, dataset collection methodology, and end-to-end connectivity empowering data flow and respective visualization. Section 3 demonstrates sensor layout in the greenhouse that is used for developing the POC. Section 4 explains the utilization of AI in sensing, along with the methodology used in this work. In Section 5, we discuss the results and analysis over utilization of RSSI to analyze crop growth. Section 6 lists the important challenges and learning drawn from deployment that can help to build more precise multidisciplinary AI solutions and outline ideas for future work. Finally, Section 7 draws the conclusions of this work.

## 2. Lorawan-Based Deployment in the Greenhouse

**Figure 3:**
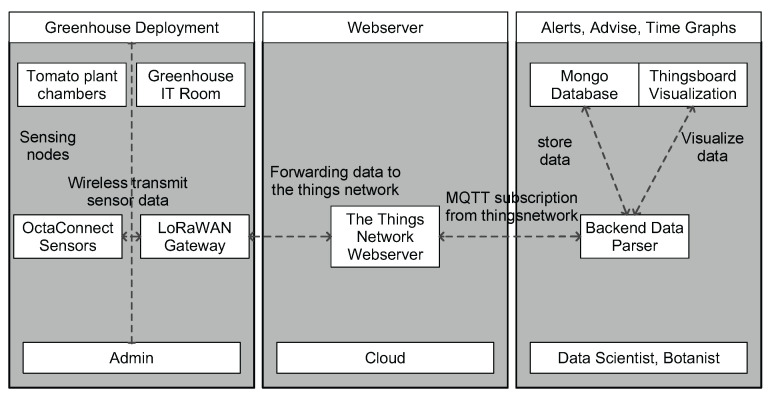
Architecture of the LoRaWAN-based sensor deployment and end-to-end data flow for a greenhouse monitoring application.

**Figure 4:**
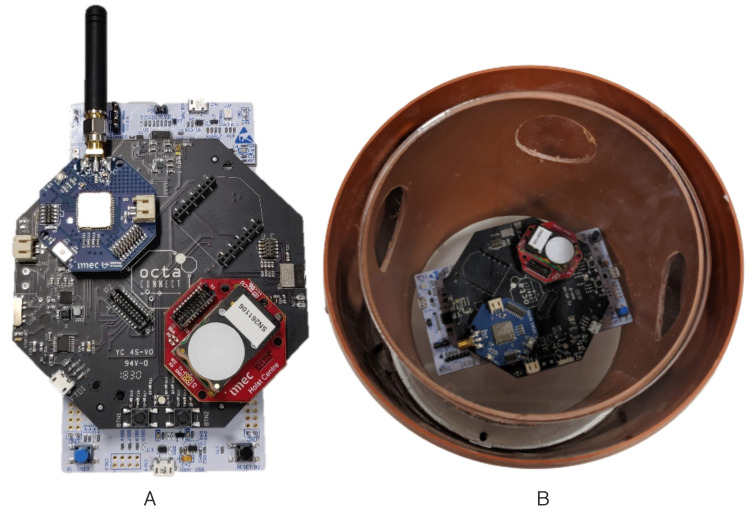
OCTA-Connect hardware modules that are used for the sensor setup. (**A**) Octa module setup with onboard temperature and humidity sensors. (**B**) Casing for the sensor setup deployed in the greenhouse with air-flow mechanism.

The experiments were conducted in a greenhouse located in PCH—Belgium, over a tomato crop. It is a research innovative center, managing 166 crop trials in the greenhouse over a period of one year. Tomato, strawberries, and bell peppers are the main crops cultivated in this greenhouse. Our experiments using WSN were conducted on a tomato crop and results were applied directly to enhance the production quality. The deployment architecture of the WSN setup in the greenhouse is shown in Figure 3. It consists of sensor and gateway deployment inside the greenhouse. We used imec’s OCTA-Connect sensor setup, leveraging LoRaWAN connectivity for the deployment as described in [25] and shown in Figure 4. The area of greenhouse ranges from 500 $m^{2}$ to 1000 $m^{2}$, thereby we used LoRaWAN technology for communication to balance out distance and keep it as a low-cost solution. Data collected by sensors are wirelessly forwarded to a Kerlink gateway installed in the PCH admin room, i.e., approximately 150 m away from the greenhouse experiment chamber. All the sensor setups have air-flow box enclosures to protect them against spray and water with a provision to stack and plug different sensors such as temperature, humidity, and light, as depicted in Figure 4. These sensors collect raw data and forward it to the things network web server, following it to our local backend using MQTT to parse, store in MongoDB database, and visualize in a custom-designed ThingsBoard dashboard. All analysis of the data, such as time graphs, alerts, etc., is performed by the user to take necessary action for crop management. Readings from sensors are received at the sampling period of 5 min.

**Figure 5:**
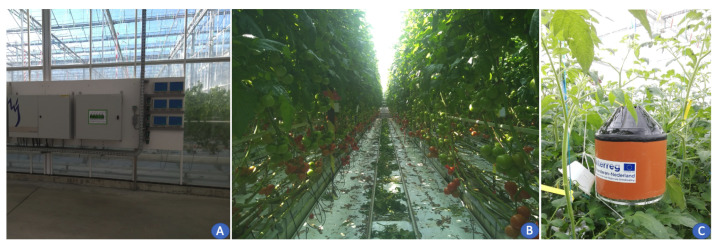
Greenhouse layout and sensor deployment in the greenhouse. (**A**) Complex infrastructure of the greenhouse. (**B**) Row of plants inside the greenhouse. (**C**) Sensor deployment in the greenhouse (air-flow (AF) boxes).

Deployment location of sensors inside the greenhouse chamber is shown in Figure 5. The first left part of Figure 5A shows complex infrastructure inside the greenhouse that can potentially impact RSSI. Next, Figure 5B shows a different row of plantations with grown tomato plants. Lastly, in Figure 5C, plants grow further and bypass the height level of sensors denoted as air-flow sensor boxes. At this point, most of the sensors are surrounded by plants. The substrate mat is located approximately 80 cm above the ground. Plants reach 3.5 m height before they are lowered again, and this is carried out for all plants to maintain even plant growth. This measurement of the plant height is taken from the substrate slab onwards. Tomatoes are sown normally by the end of January and are planted in the greenhouse using rock wool substrate by the end of March with a distance of 20 cm apart.

### 2.1. Dataset Collection Methodology

Lastly, a large dataset of LoRaWAN messages was obtained from the greenhouse. Altogether, we collected datasets from two greenhouses based in Belgium and Netherlands, respectively. For the greenhouse in Belgium, messages were received from 27 sensors with temperature and humidity data over a period from April 2020 till November 2020. A few sensors worked only till July, due to battery or hardware issues, and were removed from the field. Each sensor sent messages in a range of 24,886 to 36,250 messages, which, in total from 27 sensors, makes a huge dataset. For our second deployment, we had 19 sensors deployed from July till December, with an average of 23,575 messages from each of the sensors. Each record in the dataset has a timestamp, temperature, humidity, RSSI, and SNR value. There are a few messages that were dropped or missed due to battery replacement and can be taken care of in data cleaning before actually modeling the data. Concisely, more knowledge can be extracted from data by observing from hourly, daily, and weekly basis as a greenhouse by default has properties of stable microclimatic conditions. Another important aspect is that all messages in both the greenhouses are received by their respective dedicated LoRaWAN gateways.

### 2.2. Motivation for Joint Communication and Sensing

One of the strategies to achieve high quality of yield is to use sensors to monitor microclimate conditions in the greenhouse. There can be dense sensor deployment to assess difference in microclimate conditions, such as temperature and humidity gradients, due to solar radiation, heating, plant growth, ventilation, etc., in the greenhouse. To be able to predict behavior of plant growth in the greenhouse, we looked into sensing conditions. For the development of prediction model and learning, input variables need to be measured over a time period, for example, weekly growth of the plant, change in RSSI, temperature, humidity, etc.

**Figure 6:**
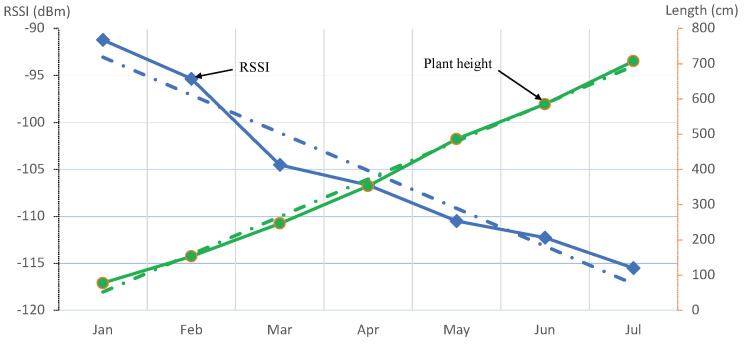
Relation of RSSI and plant growth over the period of 7 months from January to July for tomato crop.

We analyzed the data from typical deployment of a single sensor box in a tomato crop greenhouse chamber for a period of seven months. This single sensor was deployed in tomato growing chamber 1, as shown in Figure 1. The RSSI changes in a greenhouse due to several factors, such as harvesting, spraying, and regular monitoring, along with plant growth. Thereby, in order to void the impact of short-term interference, we took the average RSSI for this single sensor. For example, in different RSSI use-cases such as localization, gathered RSSI values are averaged over a sampling period to obtain the estimation [26]. We took the RSSI from our storage/dataset and took the monthly average from January till July, as shown in Figure 6. This period covered the full crop cycle from sowing season to the harvesting of crop (7 months). Altogether, a total number of 77,945 LoRaWAN messages containing temperature and humidity values of the greenhouse were received at our backend from this single sensor. Each message also had the battery level appended, which was used to foresee and change the batteries of sensors to minimize message loss. In our database, we stored these values along with RSSI, SNR, and gateway ID to obtain insights of communication link for each of the messages received. As part of regular manual measurements, PCH (greenhouse admin) took weekly measurement of the plant height. Initially, we performed knowledge discovery on data received from PCH, i.e., weekly plant height, and correlated them with received signal strengths during the same period for one sensor to inquire for any relation, if exists. The derived relation is shown in Figure 6. It depicts that with growth of the plants, there is a corresponding relational change in RSSI values. The linear analysis of plant height and RSSI data explains the phenomena of stunted growth, which can further infer possibility of plant disease, etc. With these results from one sensor, we further used another set of 27 sensors for MLP, as shown in Figure 1, for learning and predicting the plant height. This relation can be used to extrapolate events to forecast a better estimate of crop health inside the greenhouse.

## 3. Smart Agriculture Design

**Figure 7:**
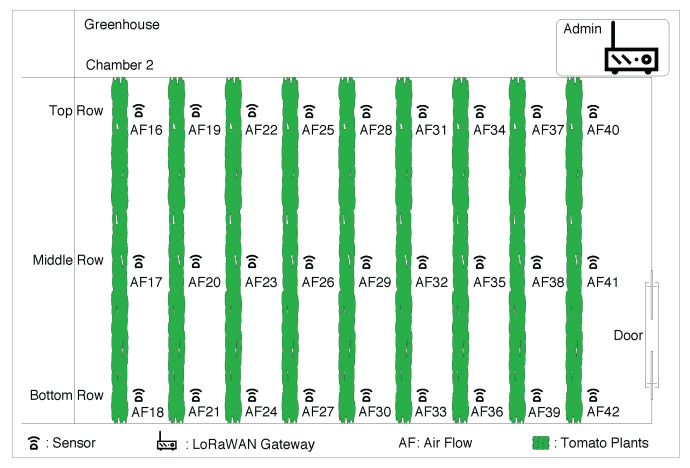
Visual representation of the chamber for growing tomato crop inside the greenhouse, along with the deployment location of sensor boxes, denoted as AF (air-flow) in each row of tomato plantation inside the greenhouse.

There are several challenges to establish an AI-based landscape sensing inside a greenhouse. Firstly, continuous flow of messages is required from multiple sensors to accommodate multipath profiles and spatial distribution analysis. Secondly, applying an AI/ML approach to learn from large dimensions of data, including features such as plant height, is complex and computationally expensive. To conceptualize the idea of AI-based greenhouse monitoring, we used the dataset of 27 air-flow (AF) sensor boxes as measurement stations on nine adjacent rows in a 3 × 9 mesh that covered the greenhouse compartment of 250 $m^{2}$, as illustrated in Figure 7. The location of the gateway was near sensor AF40 and the exit from greenhouse was near sensor AF42. All 27 deployed sensors sent temperature and humidity readings at an interval of 5 min. This experiment was carried out on the data for a period of 4 months, i.e., April to July, considering crop season and message consistency. Each of the sensors sent approximately 17,000 messages, i.e., a total of 459,000 messages were received at our backend. We also received weekly measurement of plant height over the same period from PCH. Without adding overhead in the sensing setup, we took RSSI features and plant height measurements to come up with an MLP capable of predicting the plant height and map with real-time data. For example, a grower in the greenhouse can have a prediction model that can estimate plant growth in a given week and can suggest if the plant is doing well or needs some attention concerning nutrients, potential disease, etc.

## 4. MLP Rational and Deployment

There are several types of NN, classified based on their structure, data flow, activation filter, etc. For instance, multilayer perceptron, convolutional neural network, radial basis function neural network, and recurring neural network are among types of NN that can be used as per the application requirement [27]. The application can vary between text processing, speech recognition, image analysis, translation, or a specific use case, such as our work on greenhouse monitoring. MLP is a class of feedforward artificial neural networks and is best suited for applications such as machine translation and complex classification of the data. In this type of NN, input data travel through neurons of various layers. It is a fully connected NN, as every node is connected to all the neurons of next layer. MLP neural networks were initially inspired by the biological neurons in the brain. Every neuron of an MLP network applies an activation function on top of a set of weighted inputs. Layers of interconnected artificial neurons form an MLP neural network that performs a regression on the input data. The weights are numeric values that are determined and tuned during a learning phase to minimize overall root mean square error (RMSE) of regression on a subset of the dataset, known as learning data. In our setup, there are three layers in the NN, i.e., input layer (input vector), one hidden layer, and, lastly, output layer (the predicted plant height).

The formation of an MLP set in training a supervised AI network is achieved through gathered sensor data, as discussed in the previous section for greenhouse sensing. Since we have a predefined manual set of data, gathered from the greenhouse in the form of plant heights, we used RSSI proportional to that measurement as the input feature set. The full characteristics of our MLP are given in Table 1. We used an MLP with an input size of 27. The size of the hidden layer was 54, and we used well-known log-sigmoid [28] as an activation function for this hidden layer. The output layer is composed of a single neuron, with an activation function to give a linear regression of the plant height. Error back propagation algorithm with a learning rate of 15 and momentum of 0.75 is used for the learning stage.

**Figure 8:**
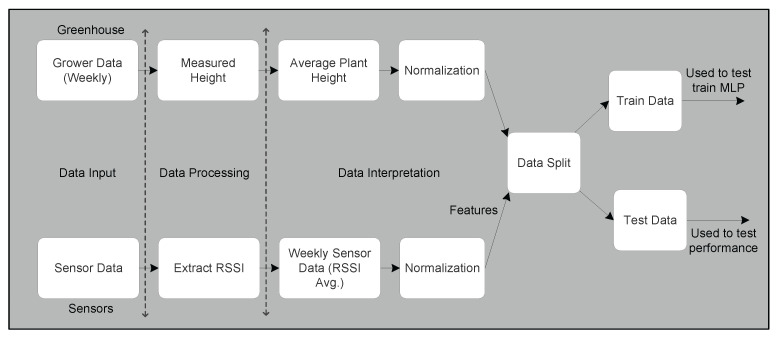
Data processing stages using inputs from sensors and greenhouse for MLP.

**Figure 9:**
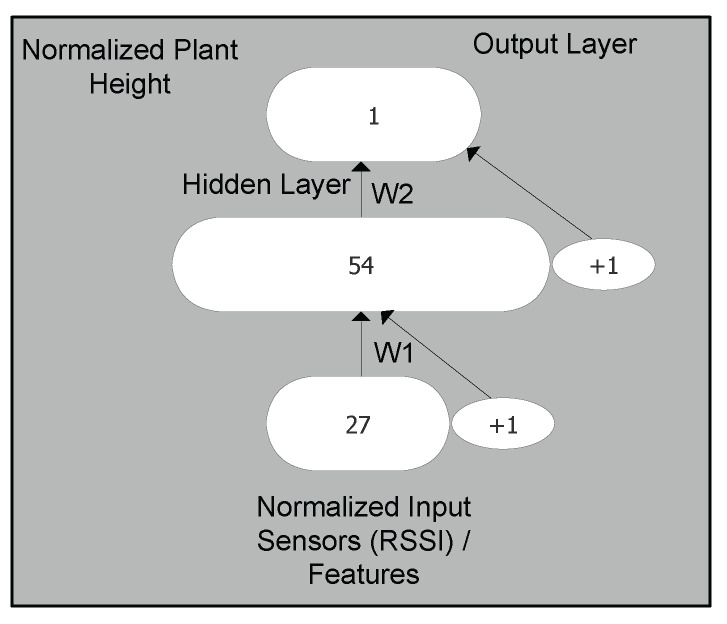
MLP architecture illustrating input, hidden, and output layer.

The processing of data is illustrated in Figure 8. It shows data usage from two separate tracks, i.e., one from the greenhouse and the other from sensors. Data collected from the greenhouse are weekly plant heights measured by the grower. We process this data to obtain an average plant height and perform normalization for better data interpretation between 0 and 1. We used a feature scaling method [29] to normalize the independent features of data, i.e., RSSI and plant height. This preprocessing of data using min–max normalization helped to scale the range in [0, 1] to have each feature contributing proportionately for learning. This data is used in the output layer of the MLP network to train and test the performance. Another set of data comes from our dataset of the greenhouse. We took this data as input to extract RSSI values for each message received at gateway as part of the data processing. Further, in order to map data from sensors to data received from the greenhouse, we performed data interpretation in a weekly format by taking an average of weekly RSSI values. The average weekly data of RSSI were again normalized between 0 and 1, as in the case of plant height to bring data uniformity. The raw RSSI data from all sensors are normalized, especially for gradient-based optimization and to accelerate the learning process, as performed in [30] for deep-learning-based indoor localization using WiFi RSSI data. These normalized average weekly RSSI data from all 27 sensors were taken as input features for MLP network. At this stage, we took the dataset from both paths, i.e., synchronized sensor and greenhouse data. This set of data acts as input to a data split function, where part of the data are used for MLP training and the rest to test performance of the MLP network. In our case, we took 70% of the data for training and the remaining 30% of the data to test the performance of the network. The MLP architecture used in this experiment is shown in Figure 9. It shows the first layer as input layer, having the data input of normalized average weekly RSSI value from the 27 sensors. The hidden layer has 54 neurons and is mapped to the output layer. Both input layer and hidden layer have one extra node, denoted as +1 in Figure 9, which is a bias node, as per MLP neural networks. There are weight matrices assigned between layers, i.e., w1 and w2. The topmost layer is the output layer, having normalized plant height for both training and test phases.

The dataset used for our MLP network is given by Equation (1), where $N^{<d>}$ is data used by the network consisting of the list of weekly RSSI values for n sensors and also weekly plant height denoted by $R_{n}^{<w>}$ and $P^{<w>}$, respectively. $T^{<d>}$ in Equation (2) is input training data of all the weekly RSSI values, from n sensors. The |.| denotes cardinality of both sets used in the network, as in Equation (3).

(1) $$N^{<d>}=\left\{G^{id}\left(R_{n}^{<w>}\right),P^{<w>}\right\},$$

(2) $$T^{<d>}=\left\{{\bar{R}}_{1}^{<w>},{\bar{R}}_{2}^{<w>},\ldots ,{\bar{R}}_{n}^{<w>}\right\},$$

(3) $$|G^{id}\left(R_{n}^{<w>}\right)\left|\;=\;\right|P^{<w>}|\;$$

## 5. Results and Analysis

Detecting plant height with the help of RSSI can be considered as an associative hypothesis problem. Such association is best seen by answering questions, such as “Does plant growth match expected week plan?”, and checking the insights of sufficient plant nutrition, disease analysis, and other supporting environmental conditions for plant growth. Answers to these matters can help the grower to analyze crop growth and take necessary actions accordingly on a weekly basis. For example, if, for a particular week, plant growth is not as predicted, then it can immediately trigger risk assessment of the crop for an early diagnosis, etc.

**Figure 10:**
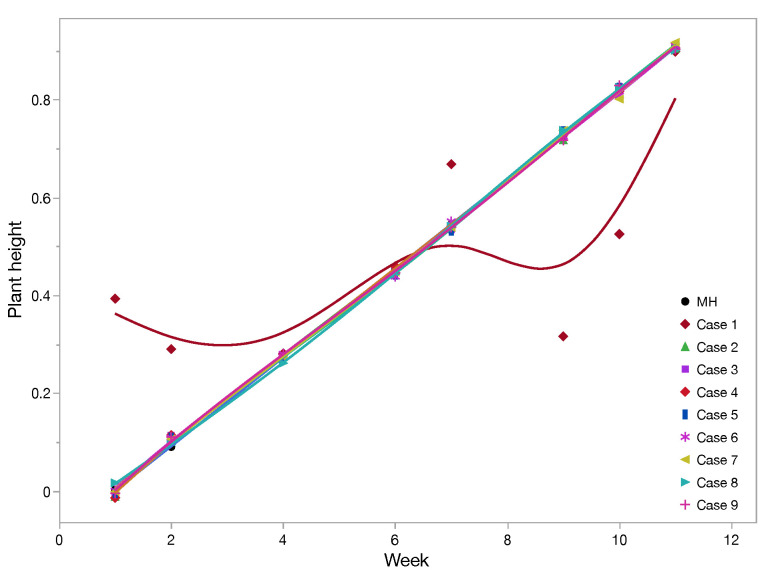
Training: Using the combination of different set of sensors as scenarios (case: Table 2) to analyze its impact on training.

**Figure 11:**
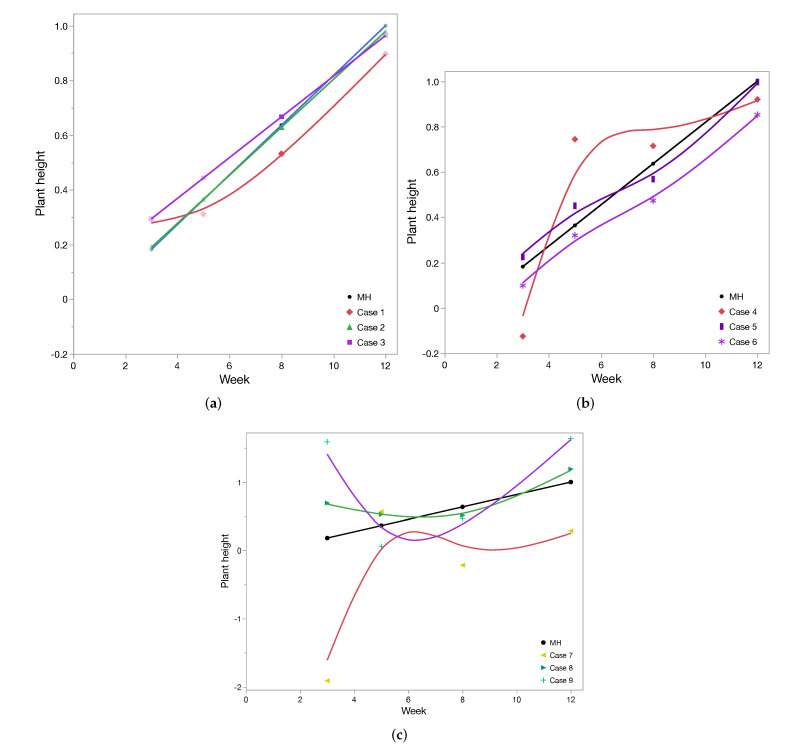
Validation: Using the combination of different set of sensors as scenarios (case: Table 2) to analyze its impact on validation in contrast to measured height (MH). (**a**) Different case with respect to number of sensors for validation; (**b**) Different case with respect to set of location-specific sensors; (**c**) Using sensors deployed in top, middle, and bottom row respectively.

A training set was constructed using data discussed in Section 4. Two suits of RSSI values from the sensors with distinct weekly values of plant heights were taken to train and access the performance of the MLP network. To this end, data were randomly divided into two subsets with a ratio of 70:30, respectively, for training and testing. The subset used to evaluate performance was never seen by the MLP before. We defined the performance in terms of RMSE. In order to test implication of different sensors in the experiment, we performed classification of different cases with respect to set of sensors and location as shown in Table 2. The first three case numbers, 1, 2, and 3, were for different sensor count; next, case numbers 4, 5, and 6 were with respect to location of sensors; and lastly, case numbers 7, 8, and 9 are three different rows of sensors. Training of all these cases are shown in Figure 10. We observed that training was not achieved properly if we used only one sensor for landscape sensing. The rest of the cases had uniform training close to measured height. Validation of all different cases are demonstrated in Figure 11a–c. We notice that the best prediction is achieved in a case of using all 27 sensors or from the set of 18 sensors deployed farthest from the gateway (case 3). By picking just nine sensors in any form from the deployed list, prediction is not good, as seen in Figure 11b. Further, more interesting results are seen in Figure 11c, where sensors close to the gateway have minimum plants in between them and the gateway, so they are not able to predict the plant height, and stand as worst-case scenario. The predicted plant heights using 27 sensors for different week numbers 3, 5, 8, and 12 are very close to the measured height. We calculated RMSE for predicting the plant height using different cases, as in Table 2. The worst case for RMSE came in using case 4, i.e., extreme left sensors farthest from the gateway, and the best result came for case 2, i.e., in using all 27 sensors. The RMSE for validating network using all 27 sensors is 10%.

**Figure 12:**
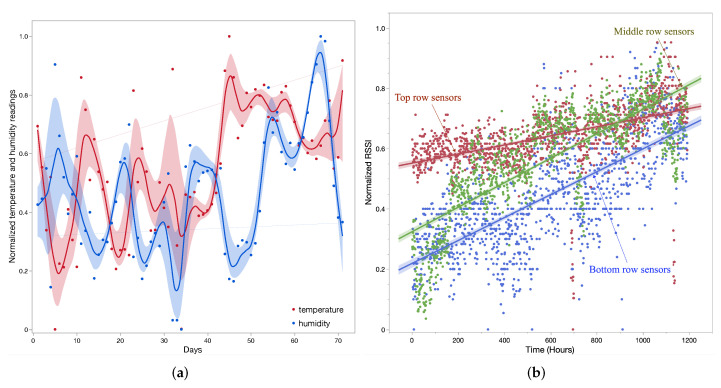
Data analysis from all sets of readings captured by the sensors in the greenhouse. (**a**) Inverse relation between temperature and humidity; (**b**) Impact on RSSI from set of sensors deployed in different rows inside the greenhouse.

From the above results, we also wanted to see change in RSSI from different sets of sensors in the greenhouse to analyze the impact of location. It is interesting from the angle of how the RSSI from different sensors varies in regard to the interference. Hypothetically, top-row sensors have the least amount of interference from plants and can communicate directly to the gateway. These sensors have no further row of plants in front of them, but just the greenhouse wall. Middle-layer sensors are surrounded by plants from all directions and plants also grow around these sensors linearly. Sensors in this row have plant growth in all directions that can directly impact the RSSI. Lastly, bottom-row sensors are farthest from the gateway and have nonuniform plant growth around them, as the bottom side is the wall and they have different growth of plants in the direction of the gateway. We normalized RSSI values for all three sets of sensors and observed the relation over a time of 1200 h in Figure 12b. As expected, it is shown that front-row sensors had minimum impact over RSSI, middle-row sensors had linear change in RSSI with the plant growth, and bottom-row sensors also had linear change in RSSI, but in a scattered manner. These scattered RSSI values for the bottom row of sensors are mainly due to more numbers of subjects causing nonuniform interference for each of the sensors.

**Figure 13:**
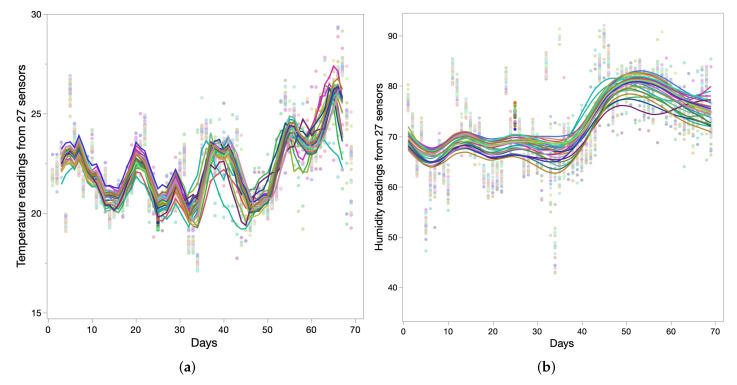
Temperature and humidity data reported by the deployed sensors in the greenhouse for a period of 70 days: (**a**) Temperature data; (**b**) Humidity data.

Every message from the sensors whose RSSI values were used had temperature and humidity measurements from the greenhouse. We analyzed these data to underpin the relation among these two values as well as their impact in the greenhouse. Normally, it is expected in the greenhouse to have a stable temperature and humidity conditions suitable for plant growth. However, we observed that there was huge variation and potential impact in readings with the outside season change. Figure 13a,b show temperature and humidity readings from 27 deployed sensors over a period of 70 days. We took the average reading for each day to illustrate the trend in the readings. It is seen in the figures that in the initial weeks of plant growth, temperature and humidity readings reported by the sensors do not vary much, as compared to readings reported in later days. This is potentially due to plant growth, i.e., as the plant grows, the temperature and humidity become more spatial throughout the greenhouse. Another important inference from temperature and humidity values is association with the outside season. During the months of March–April, the average temperature inside the greenhouse is less than 24 °C, and in June it rises above 26 °C. For crops such as tomato, it is important to have temperature range between 20 °C to 24 °C for a faster and more certain germination [31]. However, with our measurements, it is seen that temperature goes beyond the range at the later part of the crop cycle. Another paramount analysis is the inverse association between temperature and humidity, i.e., as temperature rises at the later stage of plant growth, humidity starts to drop. This inverse relation is derived by normalizing the temperature and humidity readings, as illustrated in Figure 12a.

**Figure 14:**
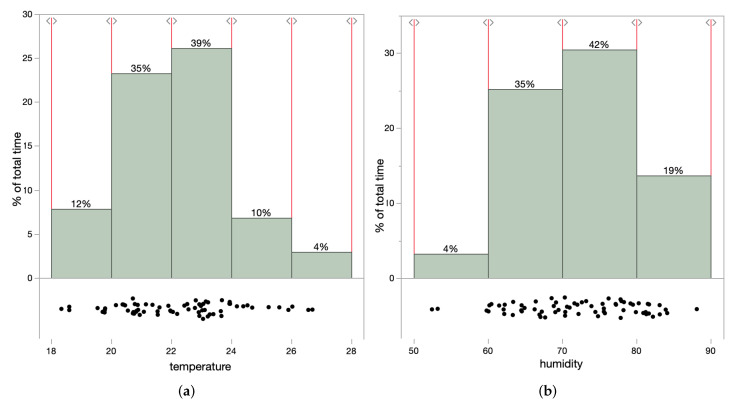
Range of temperature and humidity in different range over the total period of greenhouse monitoring. (**a**) Temperature range above 4% and below 12% of threshold, respectively; (**b**) Humidity range above 19% and below 4% of threshold, respectively, over the total time span.

There are potential tomato diseases that can spread quickly if temperature and humidity are not stable in the greenhouse. A few of the major tomato diseases and underlying reasons are listed in Table 3. It shows the impact of individual temperature and humidity, along with the combination of both. We noticed from Figure 14a,b that for a crucial percent of the total time, the range of temperature and humidity were out of the suggested boundaries.

**Figure 15:**
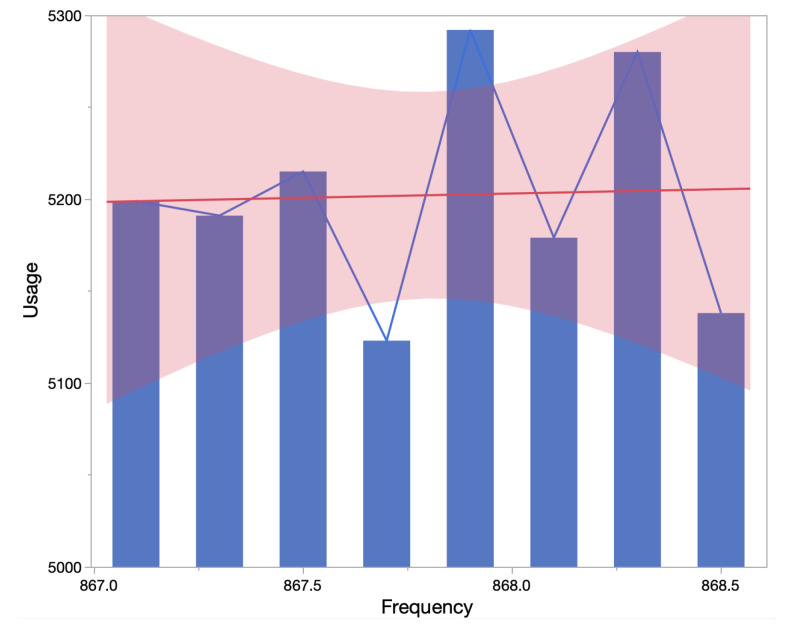
LoRaWAN channel utilization for all messages received during the experiment.

All messages sent from the sensor nodes also had appended message counter to check the number of messages lost. For each sensor node, the average number of messages lost was 103 messages, that is 0.5% approximately. This shows that message transmission from sensor node to backend via gateway was reliable, and LoRaWAN is well suited for greenhouse monitoring. In Singh et al. [32], traffic analysis and utilization percentage are calculated for the LoRaWAN network. There were a few channels with utilization of 10%, and others with 18%. However, over the period, channel utilization has become more uniform for LoRaWAN as shown in our traffic analysis demonstrated in Figure 15. We analyzed over 40,000 LoRaWAN messages and, on average, all channels had uniform utilization, with above 5000 messages per channel.

## 6. Perspective

The joint communication and sensing introduced a new perspective in the context of agriculture that includes challenges and open research questions, as discussed in this section.

### 6.1. Challenges and Limitations

**Figure 16:**
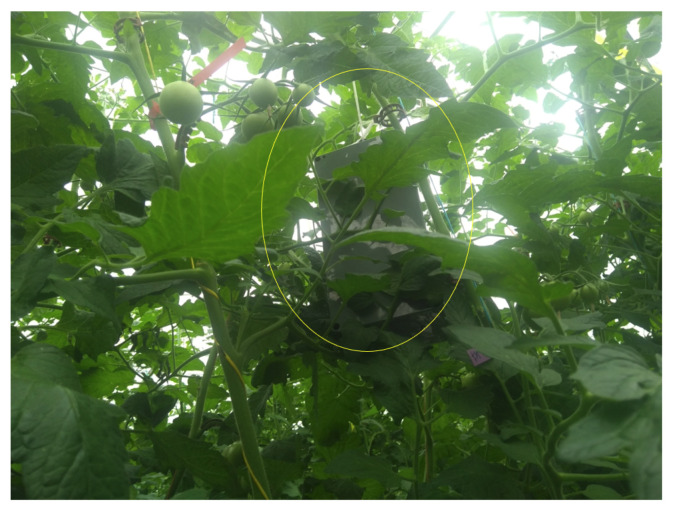
Sensors covered with plants can result in improper prediction due to the variation in RSSI.

It is crucial to consider the following limitations and factors for efficient communication-based sensing in the greenhouse:

- Radio signals can be impacted by the structure and cover material used to build the greenhouse. Inconsistent and temporal interference by different means can limit this solution.
- LoRaWAN can send a message to a different gateway that may result in different RSSI values for each message received. Thereby, tracking gateway ID is important information for using RSSI values as a feature set.
- The height of sensors from the plant needs to be uniform, such that at any moment plants should not cover sensors and maintain equal distance from the plant bed. As illustrated in Figure 16, plants can grow to the height of deployed sensors and can cover it with leaves, etc., which may potentially impact signal strength due to involved interference.
- Knowing the location of sensors can bring added value and can help to infer the location-specific plant growth. The location of the sensor can be added to the MLP network.
- Size of the greenhouse is an important aspect to be analyzed before deployment to make sure the LoRaWAN network has good coverage and an optimal number of sensors required to cover the area.
- There can be several instances where a grower or designated worker visits inside the greenhouse. Such presence of additional humans or machines may add error to the prediction model by affecting RSSI values. Recording the time slots of harvesting period or other manual work inside the greenhouse can help to refine the data prior to passing them to model.
- Greenhouses are subject to opening and closing of windows, etc., to maintain airflow, therefore the amount and periodicity of natural ventilation should be regulated or captured in records.
- Adequate plant spacing, watering, etc., resulting in condensation run-off may have inference on the signals as well.
- Lifespan of the crop as well as material used in a greenhouse may result in different signal attenuation performance.
- Apart from the mostly used polycarbonate structure, greenhouses are made up of glass as well. It is noncombustible, and with layered paned glass, it can impact the signal propagation.

Thereby, it is vital to consider the above challenges before deploying a solution in the greenhouse.

### 6.2. Future Work and Open Research Questions

The demand for food is growing exponentially with the increasing population. This ever-growing requirement for efficient and sustainable crop growth needs the support of technology such as AI for better assistance. A huge amount of data are accumulated while sensing the microclimatic features such as temperature and humidity. We can keep adding these data to the public datasets for better learning. Slowly, growers are adapting to the advancement in emerging technologies, but still struggle with questions, such as “How many sensors are required to monitor the greenhouse?”, “How can I make use of data generated from the greenhouse?”, “Are my plants growing normally?”, “Are my strategy and crop cycle optimal?”, “How can I obtain insights into plants without adding much infrastructure and cost?”, etc.

To answer a few of the aforementioned questions—a system that continuously monitors and provides crop input to the grower enabling cost reduction, acting before an issue proliferates (disease), and other crop granularities can be beneficial. With our POC, a grower can use signal strength of the regular sensor communication to predict crop growth. This is great from the perspective that growers do not need to install extra infrastructure, and it avoids the manual effort of measuring plant growth and then mapping it with expectations, as per the crop model. However, this solution can be further enriched by the inclusion of temperature and humidity values as input features for greenhouse sensing. In the work by Guidara et al. [34], the authors experimented to analyze the impact of temperature and humidity on RSSI. They concluded that there is a strong negative correlation between temperature and RSSI and there is a positive linear correlation between humidity and RSSI. Thereby, it would be interesting to include the temperature and humidity correlation as an input feature set to our MLP network for more precise information. Currently, our solution for predicting the plant height is generic for a full greenhouse chamber. Another important future work would be to consider location of sensors as an input. This will bring spatial distribution of the prediction throughout the greenhouse and can help provide location-oriented plant growth details. By the inference of location-oriented input, growers can go to a specific location with a potential anomaly to identify the cause of change in plant heights, such as due to water, start of disease, or fewer nutrients.

The current input to our MLP network does not include data of all aspects (e.g., disease and pests) and only uses signal strength of messages from the sensors. To predict crop growth in a more optimized fashion, it would be required to pass on details of the greenhouse operations to the AI solution, such as opening and closing of the greenhouse window, etc. In addition, it might be beneficial to include the details of harvesting, spraying, and other manual labor in the greenhouses in order to increase precision. Next to that, it might also be possible to predict other parameters, such as weight of the fruits and stem diameter (values that are already known to the grower by manual measurement), by regression model.

## 7. Conclusions

In the upcoming years, AI and datasets will play a crucial part in the evolution of greenhouse solutions. With that being said, adoption of AI technology in the greenhouse will not come without challenges. The greenhouse has a dynamic range of temperature and humidity, which influences crop behavior and electronic components, along with the sensors. These variations easily impact the weekly crop plans and self-learning technology. It takes time to collect data for ML methods, such as NNs, to learn crop cycles and the involved patterns. Thereby, growers adopting AI solutions may need to hold back for a seasonal crop cycle to learn intricacies for both crop and environment before producing results.

This paper describes the monitoring and collection methodology of large datasets for greenhouse monitoring using 27 sensors over a period of several months. These datasets are openly available for the global research community and can be an important tool for research in precision agriculture, and can be an efficient learning tool. This work stands as the benchmark POC on communication-based sensing using datasets. Use of an MLP neural network is carried out using RSSI from a deployed LoRaWAN-based sensors network as an input feature set for predicting plant height. By developing a POC through an MLP, we concluded that signal strength trends vary in the greenhouse over a crop period and are proportional to the plant growth. The MLP performance was evaluated for training and validating over the period of 12 weeks to predict plant height from RSSI. Results of the developed POC illustrated a good prediction of plant height with the help of RSSI values having an RMSE of 10% by using a full set of 27 sensors, and for a worst case in using just nine sensors. Further, with the help of datasets, we analyzed uniform channel utilization of LoRaWAN signals for communication and inverse relation between temperature and humidity. We also derived the impact of location of sensors over RSSI and relation with plant growth.

AI-assisted solutions for a greenhouse can potentially support growers to improve crop production. The technical setup of the greenhouse should meet certain criteria to minimize nonuniform signal attenuation, which is a major limitation of this work for empowering efficient landscape sensing. This can be majorly due to dynamic changes in infrastructure, such as from quality of material, change in layout, and bringing or removing infrastructure from crop chamber in the greenhouse. AI-based sensing using just RSSI with no added infrastructure can lower the solution cost to growers and improve crop production. 

## Figures and Tables

**Table 1:** MLP network characteristics.

Parameter	Value
Type of neural network	Multilayer perceptron (MLP)
Size of input	27
Size of layers	54
Activation function	Log-sigmoid
Activation function of last layer	Linear
Learning algorithm	Back propagation (BP)
Learning rate	15
Momentum	0.75

**Table 2:** Considering different number of sensors from the deployment and location (Figure 7) for validation.

Case No.	Sensor No.	Sensor Location
1	Only AF29	Single center sensor
2	AF16–AF42	All 27 sensors
3	AF16–AF33	18 extreme left sensors
4	AF16–AF24	9 extreme left sensors
5	AF25–AF33	9 sensors from middle
6	AF34–AF42	9 extreme right sensors
7	AF16, 19, 22, 25, 28, 31, 34, 37, 40	All top row sensors
8	AF17, 20, 23, 26, 29, 32, 35, 38, 41	All middle row sensors
9	AF18, 21, 24, 27, 30, 33, 36, 39, 42	All bottom row sensors

**Table 3:** Tomato crop disease, majorly caused due to variation of temperature and humidity [33].

Disease	Reason
Bacterial spot	Warm temperature
Bacterial wilt	High temperature and humidity
Buckeye rot	High humidity
Early blight	High humidity and mild temperature
Gray mold	High humidity and cool temperature
Late blight	Cool nighttime temperatures, and warm daytime temperatures
Leaf mold	High humidity
Septoria leaf spot	Dew and high humidity
Southern blight	High temperature
Bacterial speck	High humidity and cool temperatures

## Data Availability

The dataset presented and used in this study are openly available in Zenodo at DOI: 10.5281/zenodo.5793684; Link: https://zenodo.org/record/5793685#.YgLhgPXMK3L.
